# Genomic profiling informs therapies and prognosis for patients with hepatocellular carcinoma in clinical practice

**DOI:** 10.1186/s12885-024-12407-2

**Published:** 2024-06-03

**Authors:** Mengqi Song, Haoyue Cheng, Hao Zou, Kai Ma, Lianfang Lu, Qian Wei, Zejiang Xu, Zirui Tang, Yuanzheng Zhang, Yinan Wang, Chuandong Sun

**Affiliations:** 1 Department of Hepatopancreatobiliary Surgery, The Affiliated Hospital of Qingdao University, Qingdao, Shandong China; 2 Department of Pathology, Beijing Chaoyang Hospital, Capital Medical University, Beijing, China; 3 Software Engineering, Northeastern University, Shenyang, Liaoning China; 4 Collage of Medicine and Biological Information Engineering, Northeastern University, Shenyang, Liaoning China; 5 Department of Obstetrics and Gynecology, Peking University Shenzhen Hospital, Shenzhen, Guangdong China

**Keywords:** Hepatocellular carcinoma, Capture-based targeted sequencing, Actionable genetic alterations, Mutation landscape, Biomarker

## Abstract

Hepatocellular carcinoma (HCC) genomic research has discovered actionable genetic changes that might guide treatment decisions and clinical trials. Nonetheless, due to a lack of large-scale multicenter clinical validation, these putative targets have not been converted into patient survival advantages. So, it's crucial to ascertain whether genetic analysis is clinically feasible, useful, and whether it can be advantageous for patients. We sequenced tumour tissue and blood samples (as normal controls) from 111 Chinese HCC patients at Qingdao University Hospital using the 508-gene panel and the 688-gene panel, respectively. Approximately 95% of patients had gene variations related to targeted treatment, with 50% having clinically actionable mutations that offered significant information for targeted therapy. Immune cell infiltration was enhanced in individuals with *TP53* mutations but decreased in patients with *CTNNB1* and *KMT2D* mutations. More notably, we discovered that *SPEN*, *EPPK1*, and *BRCA2* mutations were related to decreased median overall survival, although *MUC16* mutations were not. Furthermore, we found mutant *MUC16* as an independent protective factor for the prognosis of HCC patients after curative hepatectomy. In conclusion, this study connects genetic abnormalities to clinical practice and potentially identifies individuals with poor prognoses who may benefit from targeted treatment or immunotherapy.

**Supplementary Information:**

The online version contains supplementary material available at 10.1186/s12885-024-12407-2.

## Introduction

Hepatocellular carcinoma (HCC) is a malignant and high heterogeneity tumour originating from the liver. It is the sixth most commonly diagnosed cancer and the third leading cause of cancer death worldwide in 2020 [1]. The incidence and mortality of HCC in China account for 45.3% and 47.1% of the world, respectively. The 5-year overall survival rate is currently only 14.1% [2]. The major risk factor for HCC is shifting from viral and alcoholic liver disease to obesity, type 2 diabetes, and nonalcoholic fatty liver disease [3]. The molecular pathogenesis of HCC involves the dysregulation of multiple signalling pathways, including Wnt/ß-Catenin, RAS/MAPK, PI3K/AKT/mTOR, TP53/cell cycle, IGFR, and MET, which is related to point mutations, copy number variations, epigenetic alternations, tumour suppressor inactivation and so on [4]. Hopefully, these discoveries will enable us to identify biomarkers for foretelling prognosis or responses to therapy.

The landscape of genetic alterations in HCC has a clear delineation, including the most prevalent mutations affecting *TERT* promoter (60%) [5], *TP53* (12–48%) [6–8], and *CTNNB1* (11–37%) [9]. The genetic alterations provide potential targets for treatment planning and prognostic assessment of HCC. About 25% of patients with HCC were detected potentially actionable mutations [10]. Sorafenib inhibits tumour growth and angiogenesis by targeting the RAF/MEK/ERK pathway and receptor tyrosine kinases [11]. PRI-724, a specific inhibitor targeting β-catenin, can be used to address HCC due to *CTNNB1* mutation [12]. HCC caused by TERT promoter mutation can be intervened by using targeted drugs such as GX301, Imtelstat, and GV1001 [13]. Regarding prognostic markers, *ARID1A*, *MLL*, [14] *LRP1B*, and *TP53* mutations, particularly the hotspot mutations R249S and V157F, are associated with poor prognosis for patients with HCC [15, 16]. Song et al. found that *TSC2* mutations were independently associated with early recurrence in HCC patients who underwent hepatectomy [17]. Nonetheless, these potential targets are yet to be translated into the actual survival benefits of patients due to the low mutation rates of most driver genes, no targeted drugs for oncogenic mutations, and the lack of large-scale multicenter clinical validation [13].

This study employed multigene sequencing panels targeting cancer driver genes involving key deregulated pathways in HCC, 175 drug-targeted genes, 23 immunotherapy-related genes, and 18 chemotherapy-related genes. Based on the real-world evidence from 111 patients with HCC, we aimed to determine the clinical viability and utility of genome analysis and whether patients can benefit from genomic profiling. Moreover, we identified mutations in four genes associated with survival, mutations in three other genes related to immune infiltration, and 292 novel potentially pathogenic mutations that could serve as potential targets for treatment decisions and prognostic assessment.

## Materials and methods

### Patient selection and clinical data collection

This is a retrospective study. We screened 111 HCC patients with somatic mutations detected by targeted-capture sequencing. They were treated at the Affiliated Hospital of Qingdao University between October 2015 and November 2020. The follow-up was conducted up to January 15, 2022. Postoperative histopathological examinations confirmed clinical diagnoses. Clinicians gathered clinical data on the progression of the condition (Table S1). All patients were treated surgically. The extent of surgical resection is shown in Table S1. The study was authorized by the Ethics Committee of the Affiliated Hospital of Qingdao University (approval no. QYFYWZLL27327). The informed consent form was offered and signed by each patient. The experiment complied with the official key recommendations of the National Health and Family Planning Commission of China.

## DNA extraction, library construction and sequencing

DNA was isolated from tumour tissue samples and whole blood samples (as normal controls) by QIAamp Fast DNA Tissue Kit and QIAamp DNA Blood mini Kit (QIAGEN), respectively. The concentration of DNA was determined using qubit fluorometry, and the integrity and purity were evaluated using agarose gel electrophoresis and the Qubit 2.0 fluorimeter (Thermo Fisher, USA). The targeted DNA sequence was then enriched and captured by two custom sequence capture probes (Nimblegen, USA) that targeted 7708 exons of 508 cancer-related genes and 10,176 exons of 688 cancer-related genes, respectively. Sequencing was performed on the MGISeq-2000 platform with a coverage depth of 1000 × for tumour tissue and 400 × for blood (MGI, Shenzhen, China).

The specific target gene list is in Table S2. There are 850 targeted genes captured by 688 and 508-gene panels, including 345 shared genes, 343 genes specific in the 688-gene panel, and 163 genes specific in the 508-gene panel. In the 508-gene panel, there were 135 genes involved in tumour signalling pathways, 89 associated with targeted therapy, and 16 associated with immunotherapy, 12 of which were associated with both targeted therapy and immunotherapy. The 688-gene panel included 452 genes involved in tumour signalling pathways, 11 associated with chemotherapy, 165 associated with targeted therapy, and 22 associated with immunotherapy, 15 of which involved both targeted therapy and immunotherapy.

## Sequencing data analysis

SOAPnuke [18] was used to remove adapters and filter low-quality reads after obtaining raw sequencing data. Using bwa-mem2 (https://github.com/bwa-mem2/bwa-mem2) [19], clean reads were mapped to the human reference genome (hg38). GATK (v 4.1.9.0) [20] was used to eliminate duplicates, identify somatic variants, and filter variants. The assessment of clinical importance and the prediction of the functional impact of sequence variants were done using ANNOVAR (http://www.openbioinformatics.org/annovar/) [21]. Somatic variants were filtered based on the following criteria: i) variants with allele depth < 10 were excluded; ii) variants with allele frequencies < 0.1 were excluded; and iii) variants with population frequencies > 1% were excluded from the further investigation based on the Exome Aggregation Consortium dataset (ExAC http://exac.broadinstitute.org), 1000 Genomes Project (http://www.1000genomes.org/) [22]. ESP6500SI-V2 and avsnp150 databases. Additionally, actionable mutations were identified using OncoKB (http://oncokb.org) [23]. HCC driver genes were identified by IntOGen [24]. TIMER (https://timer.comp-genomics.org/) [25] database offered tumour immune infiltration analysis.

## Mutation statistics and visualization

Detailed information about mutations, including their features, distribution, and enrichment in oncogenic signalling pathways, was compiled and visualized using the R package maftools (version 2.8.05) [26]. We measured overall survival (OS) from the date of the first clinic visit to the last follow-up or death. Survival analysis was visualized using the R package survival (version 3.3.1) and survminer (version 0.4.9), using R package "jskm" to make landmark analysis. We used the Oviz-Bio platform to landscape the mutation type, mutated gene, mutation frequency, and clinical data about the patient [27]. Associations between driver genes and clinical features and the difference between the rates of affected cases in the TCGA cohort and this cohort were investigated using Fisher's exact test or the χ^2^ test. *P* less than 0.05 was deemed significant.

## Results

### Clinical characteristics of the patients with HCC

One hundred and eleven patients with HCC were included in this study (17 in female, 92 in male and two unknown). The median age was 53.5 years (range 33–78). According to TNM staging, the majority of patients (44.14%, 49/111) were in stage T1b. Over 40% of patients had small lesions with 2-5 cm tumour diameters, while 14.41% had large lesions with diameters greater than 10 cm. Lymph node metastasis was common in HCC and a key step in tumour metastasis. In our study, over one-quarter of patients developed lymph node metastasis. Furthermore, 30 patients (27.03%) experienced relapses, with the most common site of recurrence being intrahepatic (50%, 15/30). Over 85% of patients had one or more risk factors, including 88 patients with hepatitis B virus infection, 31 drinkers, and 17 with diabetes.

Concerning clinical indicators, the ASL/ALT ratios in serum were less than 0.8 in 29 patients and greater than 1.5 in 14 patients. The AFP level of 56 patients was above 25 ng/ml, and the CA19-9 level of 25 patients was more than or equal to 37 U/ml. In addition, most patients had normal CEA and CA125 levels (Table 1 and Table S1).

**Table 1:** Clinical characteristics of 111 patients with Hepatocellular carcinoma

Clinical characteristics (*n* = 111)	
**Age-years**	
Median	53.5
Range	33–78
**Sex**	
Male	92 (82.89%)
Female	17 (15.31%)
Unknown	2 (1.80%)
**TNM Stage**	
T0	1 (0.90%)
T1	59 (53.15%)
T2	15 (13.51%)
T3	13 (11.71%)
T4	19 (17.12%)
Unknown	4 (3.60%)
**Tumor Diameter**	
0-2 cm	13 (11.71%)
2-5 cm	48 (43.24%)
5-10 cm	31 (27.93%)
> 10 cm	16 (14.41%)
Unknown	3 (2.70%)
**Lymph Node Metastasis**	
Yes	30 (27.03%)
No	79 (71.17%)
Unknown	2 (1.80%)
**Tumor Recurrence**	
Yes	30 (27.03%)
No	79 (71.17%)
Unknown	2 (1.80%)
**With Diabetes**	
Yes	17 (15.32%)
No	92 (82.88%)
Unknown	2 (1.80%)
**Smoker**	
Yes	34 (30.63%)
No	75 (67.57%)
Unknown	2 (1.80%)
**Drinker**	
Yes	31 (27.93%)
No	78 (70.27%)
Unknown	2 (1.80%)

## The spectrum of somatic mutations in genes

**Fig. 1:**
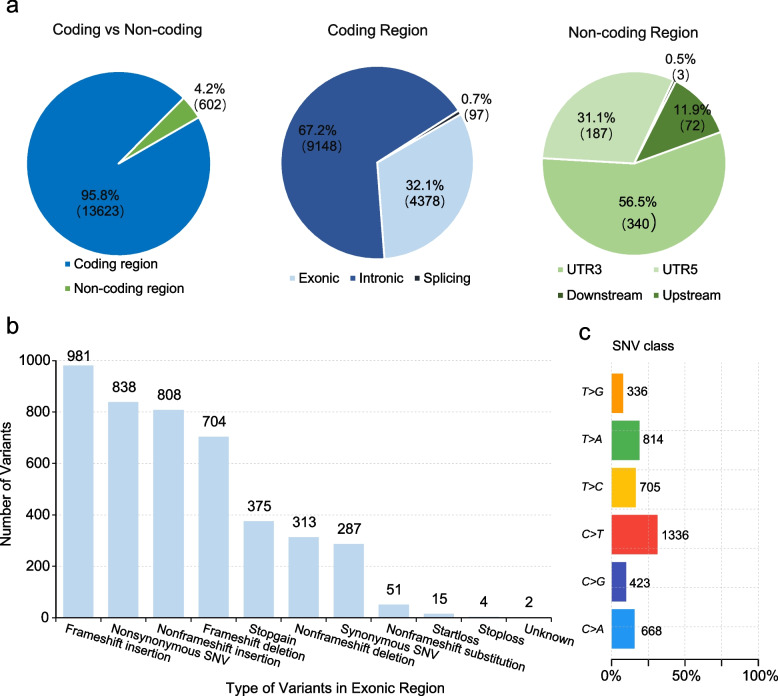
Results of variants calling in 111 patients with liver cancer. **a** Distribution of mutant sites in the coding and non-coding regions of DNA. **b** Types and numbers of variants. **c** Numbers of each SNV class

Of these 111 patients, 86 and 25 were detected mutations in 688 genes and 508 genes, respectively. In total, we detected 14,225 somatic mutations in all patients, including 1,125 SNVs, 1,789 insertions, and 1,017 deletions. Most mutations were located in the coding region, 32.1% in exonic regions, and 0.7% in splicing regions (Fig. 1a). The two most common types of mutations were frameshift insertion and nonsynonymous SNV (Fig. 1b). For SNV, C > T was the major mutant form (Fig. 1c). In addition, there were 287 synonymous mutations, 9,148 variants in the intronic region, and 602 variants in the non-coding region, which were filtered out in the following analysis.

**Fig. 2:**
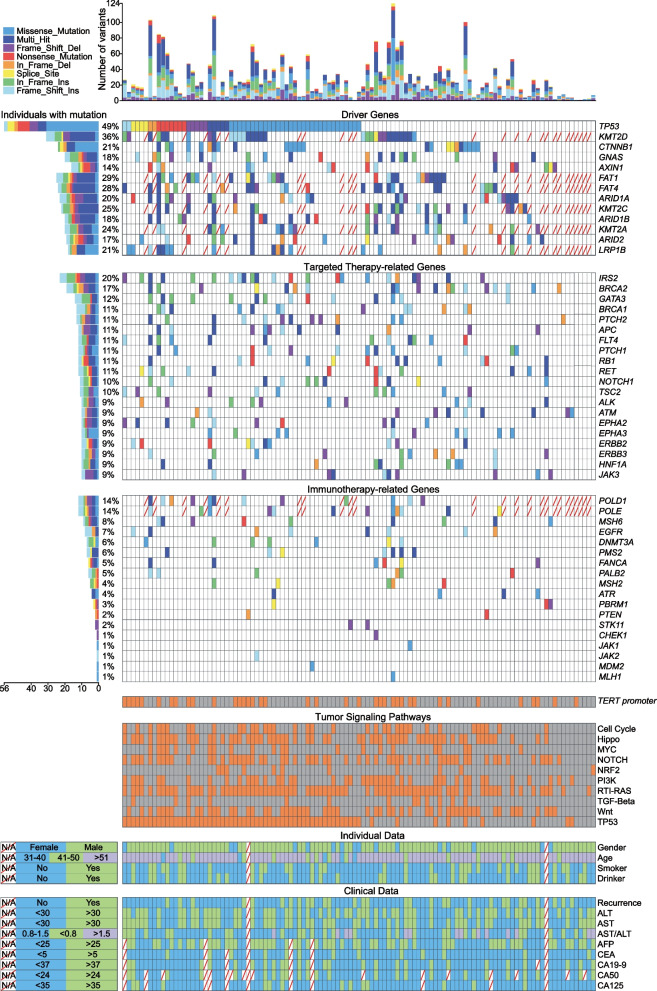
The landscape of frequently mutated genes of liver cancer. Significantly mutated genes in 111 patients. Above, the histogram shows the number of variants of each patient. Left, the percentages of patients with mutations. Diagonally indicated the information that was not available. Different colours correspond to different types of mutations. Variants annotated as Multhit_Hit are those genes that are mutated more than once in the same sample

**Fig. 3:**
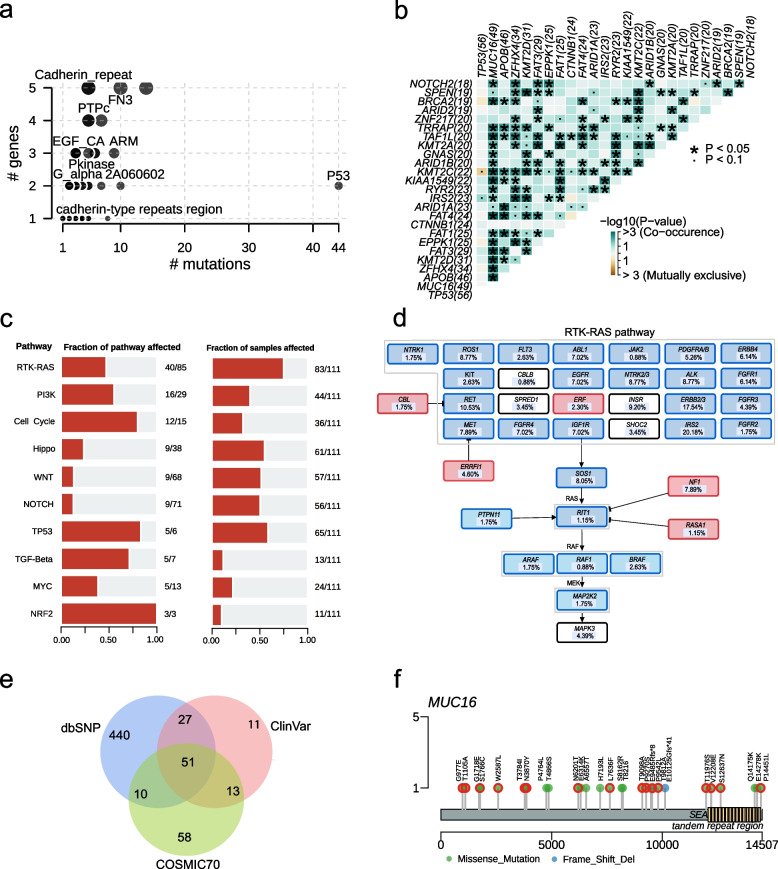
Clinical implications of mutations and domain and pathway enrichment analysis. **a** Frequently mutated Pfam protein domains in liver cancer. The bubble size is in proportion to the number of genes containing prominent display domains. **b** Somatic gene interactions. **c** Enrichment of known oncogenic signalling pathways. **d** Mutated genes in the RTK-RAS pathway. Tumor suppressor genes are in red, and oncogenes are in blue. **e** Venn plot of database-registered variants. **f** Location of the MUC16 mutations schematic. Red circles highlight novel missense mutations

We identified 57 driver genes mutated in 111 patients in this study. About 34% (38/111) of patients harboured *TERT* promoter mutations. The frequently mutated driver genes were *TP53* (50.45%, 56/111), *KMT2D* (36.05%, 31/86), *FAT1* (30.23%, 26/86), *FAT4* (29.07%, 25/86), and *KMT2C* (26.74%, 23/86; Fig. 2). The P53 structural domain was affected most frequently (Fig. 3a). As driver genes, *TP53* and *CTNNB1* had no interactions with other genes, while Histone-lysine N-methyltransferase 2 (*KMT2*) family genes had a synergistic effect with the *FAT* gene family, *ARID1A/B*, and *GNAS* (Fig. 3b). Except for these driver genes, the top five high-frequency mutation genes were *MUC16* (56.98%, 49/86), *APOB* (52.33%, 45/86), *ZFHX4* (39.53%, 34/86), *FAT3* (26.13%, 29/111), and *EPPK1* (22.52%, 25/111; Figure S1). Epigenetic modifiers, such as *ARID1A* (21.62%, 24/111), *ARID2* (17.12%, 19/111), and *MLL* genes, were also recurrently altered.

## The spectrum of somatic mutations in driver pathways

RTK-RAS (72.81%), TP53 (57.01%), Hippo (53.51%), Wnt/ß-Catenin (50%), NOTCH (49.12%), PI3K (38.60%), and Cell Cycle (31.58%) were activated frequently (Figs. 3c and S1). We found 40 mutated genes involved in the RTK-RAS oncogenic signalling pathway, with five of the mutated genes being tumour suppressors and 35 being oncogenes. Oncogenes *IRS2* (20.72%, 23/111), *RET* (10.81%, 12/111), and tumour suppressor genes *NF1* (8.11%, 9/111) frequently mutated in the RTK-RAS pathway (Fig. 3d). The mutations in *KMT2D*, *CTNNB1* (21.62%, 24/111), *GNAS* (18.92%, 21/111), and *AXIN1* (14.41%, 16/111) affected Wnt/ß-Catenin pathway and mutations in *TP53*, *ATM* (9.01%, 10/111), *RB1* (11.71%, 13/111), *CDKN2A* (8.11%, 9/111), and *CDKN1A* (5.41%, 6/111) altered cell cycle control (Figure S2). The oxidative stress pathway was altered in 9.65% of patients with mutations in *NFE2L2*, *KEAP1*, and *CUL3*.

## Clinical implications of mutations

We used the CLINVAR, dbSNP, COSMIC, and OncoKB databases to analyze the clinical significance of mutations. In our study, 528, 132, and 102 functional and meaningful variants have been registered in the dbSNP, CLINVAR, and COSMIC databases, respectively (Fig. 3e). More importantly, 92.98% of patients had variants in targeted therapy-related genes (Fig. 2). Among them, 110 variants of 20 genes in 55 patients were reported as drug targets. In other words, 49.55% (55/111) of patients in this study had potentially actionable genomic alterations that required further clinical trials for HCC. For example, frameshift indels and stopain mutations in *ARID1A* and *TSC1/2* were the targets of EZH2 inhibitors (Tazemetostat and GSK126) and mTOR inhibitors (ABI-009 and Everolimus), respectively. The nonsynonymous SNV, G3145C, in exon 21 of *PIK3CA* was the target of PIK3 inhibitors (Table S3). According to the follow-up results, 15 patients received targeted therapy, immunotherapy, and/or chemotherapy due to tumour recurrence (see Table S1 for treatment options). Three patients were found to carry *TP53* mutations associated with sorafenib resistance. This provides evidence for the ultimate selection of lenvatinib. One patient who received sorafenib possessed *CCND1* mutation that can result in sensitivity to sorafenib. Moreover, genotype CT of rs11598702 in *NT5C2* suggested that one patient with this genotype may have a lower risk of toxic side effects with the use of gemcitabine. Ultimately, this patient also received chemotherapy with gemcitabine. The genetic testing results provided follow-up medication reference evidence for one-third of these 15 patients.

Three gene mutations have been found to be associated with immune infiltration. Based on the TIMER database, patients harbouring *TP53* mutations had higher levels of B cells (*P* = 0.039) and macrophages (*P* = 0.023; Figure S3). In contrast, patients harboring *CTNNB1* and *KMT2D* mutations had lower levels of CD8^+^ T cells (*P* = 0.003 for *CTNNB1*; *P* = 0.004 for *KMT2D*), macrophages (*P* < 0.001; *P* = 0.004), neutrophils (*P* < 0.001; *P* = 0.002) and dendritic cells (*P* = 0.004;* P* = 0.007).

We performed pathogenic mutation prediction using 21 algorithms. For the 631 novel variants, at least five algorithms predicted that 292 variants were deleterious, of which we detected the most novel pathogenic mutations in *MUC16*, following *DNMT3A, UPF1, COL11A1*, and *BIRC3* (Tables S4 and S5). The variants in *MUC16* included 26 missense mutations and two frameshift deletions, of which 57.14% were novel pathogenic mutations. Most novel pathogenic mutations were located outside the SEA and tandem repeat region structural domains (Fig. 3f).

## Prognostic implications of genomic and clinical features

**Fig. 4:**
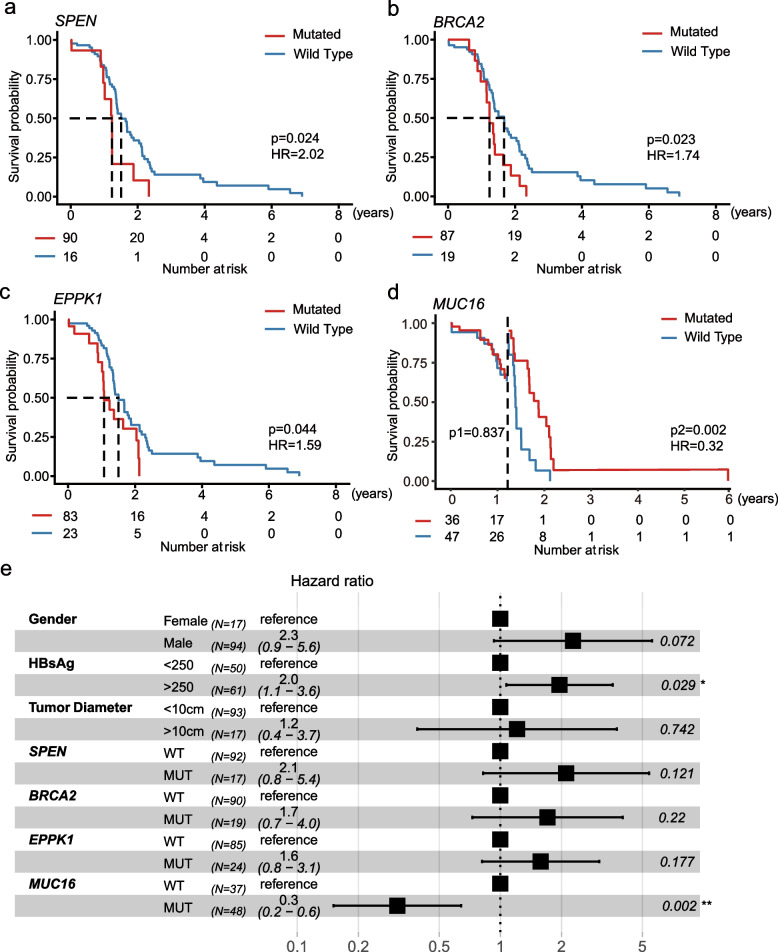
Survival analysis of mutated genes. **a**-**d** Survival analysis of *SPEN*, *BRCA2*, *EPPK1*, and *MUC16*. The red line indicates the mutated group, and the blue line indicates the wild-type group. **e** Multivariate analysis of clinical prognostic factors of HCC. Log-rank test. HR: Hazard ratio

We compared survival between patients with or without mutations in genes. The median follow-up of 111 patients was 14.8 (IQR 0.1–84.0) months. We explored the relationship between genes with mutation frequencies greater than 15% and survival and found that alterations in four genes correlated with a poor or good prognosis. Patients harboring *SPEN* (18.3 vs. 15.0 months; Log-rank test, *P* = 0.024; Fig. 4a), *BRCA2* (20.3 vs. 15.1[months; *P* = 0.023; Fig. 4b), and *EPPK1* (18.3 vs.13.1 months; *P* = 0.044; Fig. 4c) mutations had a shorter OS, while patients harboring *MUC16* (18.7 vs. 15 months; *P* = 0.002, after landmark; Fig. 4d) mutations had a longer OS. Mutations in *BRCA2* (HR = 1.74), *EPPK1* (HR = 1.59), and *SPEN* (HR = 2.02) were risk factors for patients with HCC, while *MUC16* mutation (HR 0.32) was a protective factor.

Moreover, we conducted univariate analysis considering clinical factors (e.g., gender, age, tumour size, HBsAg) and genes with mutation frequencies > 15%. Univariate analysis revealed that *SPEN* (*P* = 0.028), *BRCA2* (*P* = 0.026), *EPPK1* (*P* = 0.047), and *MUC16* (*P* = 0.022) mutations were associated with the prognosis of HCC after hepatectomy. No clinical factor was found to be associated with prognosis. Next, we included the above four genes, as well as clinical factors (i.e., gender, HBsAg, and tumor size) in our multivariable regression analysis based on clinical significance and previous literature research findings. Positive HBsAg (HR = 2.3, 95% CI 1.2–4.6) was a risk factor for the prognosis of HCC patients after curative hepatectomy. Otherwise, mutant *MUC16* (HR = 0.2, 95% CI 0.1–0.5) was a prognostic protective factor (Fig. 4e).

## Associations between driver genes and clinical characteristics

We explored the relationship between driver genes and clinical characteristics. *LRP1B* mutations were more common in smokers (55.88% vs 44.12%; χ^2^ test, *P* = 0.03). Also, *LRP1B* mutations appeared to be associated with tumor diameter, which was more likely to be greater than 10 cm in patients with *LRP1B* mutations (68.75% vs. 31.25%; *P* = 0.01; Table S6).

## Discussion

Based on the real-world evidence from 111 Chinese patients, this study enhanced our comprehension of genome analysis's clinical viability and utility in HCC. Approximately 95% of patients had mutations in driver genes and/or pathways in HCC and 48.25% potentially actionable alterations, which yielded valuable information for targeted therapy or immunotherapy. *TP53*, *CTNNB1,* and *KMT2D* mutations were related to immune cell infiltration. *SPEN*, *EPPK1*, *BRCA2*, and *MUC16* mutations were associated with OS. More importantly, we identified mutant *MUC16* as an independent protective factor for the prognosis of HCC patients after curative hepatectomy.

We revealed hotspot mutations in 111 Chinese patients with HCC, and the genomic mutation frequency of *CTNNB1* (21.6% vs. 22.6%), *AXIN1* (14.4% vs. 13.7%), *RB1* (11.7% vs. 11.9%) in our cohort was not significantly different from previous reports [28]. However, the mutation frequency of *TP53* (50.5% vs. 56.5%) and *TERT* (34% vs. 45.2%) in our cohort are lower to the cohort of Wang et al. [28]. We deduced that the difference in the number of patients with HBV infection may be one of the reasons. Some studies have reported that the mutation frequency of *TP53* was higher in HCC caused by HBV infection than those without HBV infection [28, 29]. In the cohort of Wang et al., 84.8% (140/165) of patients were positive for hepatitis B surface antigen (HBsAg), while in our cohort, only 54.1% (60/111) of patients were positive for HBsAg. Moreover, Wang et al. counted more mutation types than us, including gene amplification and fusion/rearrangement. Another reason might be that the criteria for patient enrollment are different. We screened HCC patients with somatic mutations and excluded patients without somatic mutations detected by targeted-capture sequencing. This might lead to a change in the frequency of gene mutations.

The immunological analysis revealed that mutations in *TP53* were related to the level of immune infiltration. A report showed that HCC patients with mutant *TP53* had significantly macrophage infiltration higher than those with wild-type *TP53* [30], which coincided with our results. Loss or alteration of p53 caused by *TP53* mutations can regulate the recruitment and activation of immune cells, resulting in the suppression or evasion of anti-tumor immune responses [31, 32]. *TP53* mutants can reprogram macrophages to tumour-associated macrophages (TAMs) [33] and were found to relate to the infiltration of TAMs into primary tumours [34]. One possible mechanism is that *TP53* mutants lead to increased expression of the chemokine CCL2. CCL2, through the CCL2-CCR2 signalling axis, recruits TAMs to the tumour area [34, 35]. Given the profound impact of the *TP53* status of the cancer cell on the immune response, previous studies have found that *TP53* mutations have the potential to serve as a predictive factor in guiding anti-PD-1/PD-L1 immunotherapy [36, 37]. Because *TP53* mutation significantly increased the expression of PD-1 and PD-L1. These studies focus on lung adenocarcinoma. High expression of PD-1 or PD-L1 has been consistently identified as a reliable predictor of a positive response to immunotherapy in various types of cancer. However, the association between *TP53* and PD-L1 expression varies among cancer types [38, 39]. Indeed, no definitive biomarkers have been identified to predict the efficacy of immunotherapy in HCC. Studies on PD-L1 expression in HCC are limited or have limited clinical value due to their low occurrence frequencies. The positive rate of PD-L1 expression in HCC tumour cells ranges from 10 to 20% [40], but objective responses have been observed in PD-1 monotherapy regardless of PD-L1 expression [41, 42]. Therefore, more comprehensive and in-depth research is needed to determine whether *TP53* mutants can serve as biomarkers for immune therapy in HCC.

Approximately 25% of potentially actionable mutations are found in HCC [6]. Unfortunately, the most prevalent drivers and trunk mutations, such as *TERT* promoter, *AXIN1*, and *TP53* mutations, are currently undruggable [43]. Nevertheless, recent studies have shown that there are already relevant, targeted drugs in Phase I to III trials [44]. For instance, *CTNNB1* mutation-blocking drugs are expected to be useful for precision medicine [44]. A Japanese early clinical experience explored the effect of Atezolizumab plus Bevacizumab (ATZ/BV) in HCC patients harbouring *CTNNB1* Mutation and found that ATZ/BV might improve the immunosuppressive tumour microenvironment caused by *CTNNB1* mutation [45]. Patients harbouring *CTNNB1* mutations are mainly manifested as immune rejection in the previous study [46] and our study. Thus, 25 patients harboring *CTNNB1* mutations in our study might use ATZ/BV to improve immunosuppression. Additionally, Lim et al. conducted a phase II clinical study on treating RAS-mutant HCC using refametinib or refametinib plus sorafenib, which has shown promise [47]. This provided new potential treatment options for RAS-mutant HCC patients.

Notably, we found that mutant *MUC16* was an independent protective factor for the prognosis of HCC patients after curative hepatectomy. *MUC16*, encoding CA125, is the second most commonly mutated gene in HCC and has the most novel potential pathogenic variants in our study. Mutant *MUC16* was also found to result in a better prognosis in gastric cancer and low-grade glioma [48–50]. The mechanisms underlying the favourable prognosis associated with *MUC16* mutations remain unclear. In gastric cancer research, the group with MUC16 mutations showed increased infiltration of tumour-killing cells and decreased presence of immunosuppressive cells [48]. The infiltration of immune cells may significantly contribute to a better prognosis. However, we did not observe any differences in immune cell infiltration between the *MUC16* mutation group and the wild-type group in our study on HCC. The mechanisms by which *MUC16* mutation leads to a better prognosis may vary across different tumours. Therefore, *MUC16* mutations may assist in HCC prognosis and should be further studied in this tumour type. Moreover, we observed that positive HBsAg was a risk factor for prognosis in multivariable analysis, but HBsAg did not show a significant association with prognosis in univariate analysis. A possible reason is that the effects of other factors are eliminated through multivariable analysis, revealing the independent effect of HBsAg on prognosis.

There are several limitations to our study. Firstly, targeted sequencing cannot detect changes in genes excluded from the assay, structure variation, and HBV/HCV integration. Secondly, sampling a single site cannot represent the whole tumour since HCC is highly heterogeneous. Thirdly, this study should be continued to collect more information on postoperative treatment and patient survival to link drug response and prognosis with molecular profiles. Fourthly, the average follow-up time of this study was not long enough (slightly over one year) to assess persistence of the impact of mutations. Despite these limitations, we identified novel potential immunotherapy efficacy and prognosis predictors.

Linking genomic alterations to clinical practice can identify patients who are likely to benefit from targeted therapies or immunotherapy and have a poor prognosis. We hope that our findings will make routine genetic testing more accessible in clinical practice and a research context.

### Supplementary Information

Additional file 1: Figure S1. Mutated genes in the TP53 pathway. Figure S2. The landscape of frequently mutated genes and chemotherapy-related genes in 111 patients. Figure S3. Association of immune infiltration with mutant genes.

Additional file 2: Table S1. Clinical information of 111 patients with Hepatocellular carcinoma. Table S2. List of the targeted genes. Table S3. Overview of the clinically actionable genomic alterations. Table S4. List of genes that have been detected by at least five software programs with new deleterious mutations. Table S5. The pathogenicity prediction results of the variants. Table S6. Thirteen mutation statuses stratified by clinical characteristics.

## Data Availability

The dataset(s) supporting the conclusions of this article is(are) available in the China National Center for Bioinformation (CNCB) repository, [unique persistent identifier and hyperlink to dataset(s) in https://ngdc.cncb.ac.cn/gvm/getProjectDetail?project=GVM000754].

## Abbreviations

HCC: Hepatocellular carcinoma
OS: Overall survival
SNV: Single nucleotide variants
IQR: Interquartile range
HR: Hazard ratio
ATZ/BV: Atezolizumab plus Bevacizumab
HBV: Hepatitis B virus
HCV: Hepatitis C virus
