# Supplementary material for: Genomic profiling informs therapies and prognosis for patients with hepatocellular carcinoma in clinical practice
Source: BMC Cancer. 2024 Jun 3;24:673. doi: 10.1186/s12885-024-12407-2 (PMC11145829; doi:10.1186/s12885-024-12407-2)
Supplement: Supplementary file 1 — Additional file 1: Figure S1. Mutated genes in the TP53 pathway. Figure S2. The landscape of frequently mutated genes and chemotherapy-related genes in 111 patients. Figure S3. Association of immune infiltration with mutant genes. [file 12885_2024_12407_MOESM1_ESM.docx]

**Supplementary Figures**


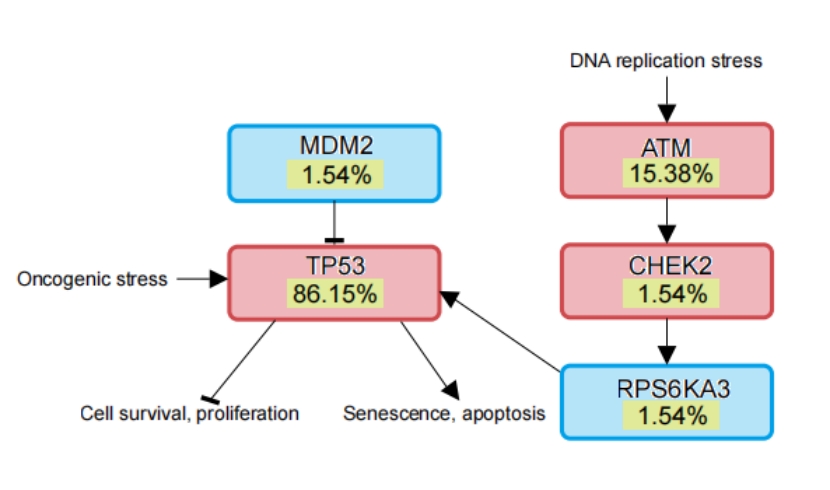


**Figure S1.** Mutated genes in the TP53 pathway. Tumor suppressor genes are in red, and oncogenes are in blue.


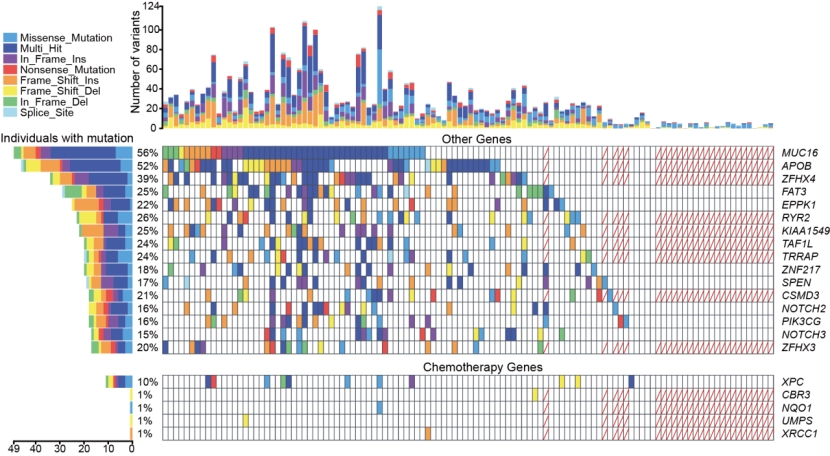


**Figure S2.** The landscape of frequently mutated genes and chemotherapy-related genes in 114 patients. Above, the histogram shows the number of variants of each patient in the corresponding panel. Right, the percentages of patients with mutations.


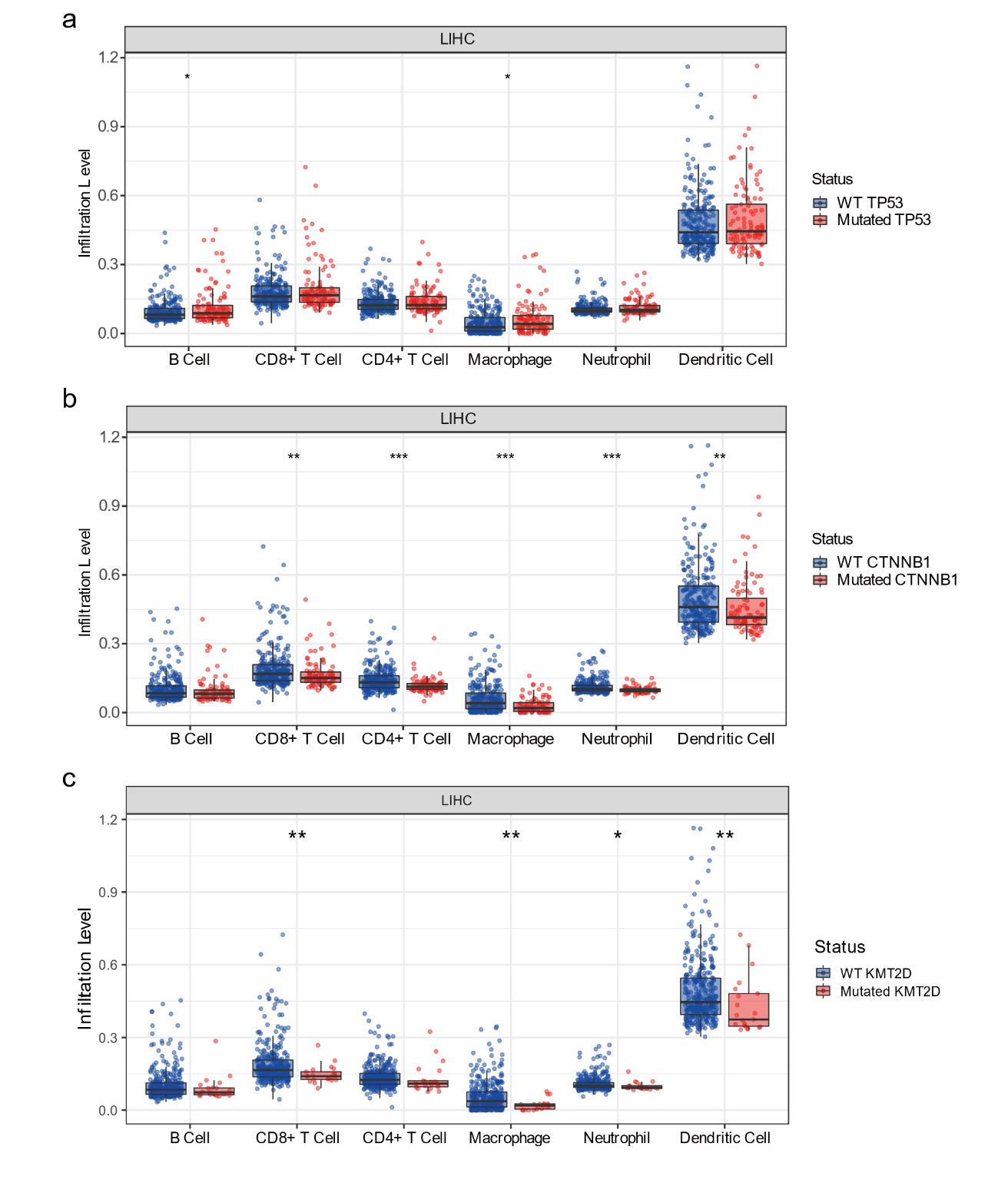


**Figure S3.** Association of immune infiltration with mutant genes. (a) Comparison of immune infiltration between *TP53* mutated and wild-type samples. (b) Comparison of immune infiltration between *CTNNB1* mutated and wild-type samples. (c) Comparison of immune infiltration between *KMT2D* mutated and wild-type samples. WT: wild type; **P* < 0.05; ***P* < 0.01; ****P* < 0.001.
